# Language-switching and retrieval-based learning: an unfavorable combination

**DOI:** 10.3389/fpsyg.2023.1198117

**Published:** 2023-07-26

**Authors:** Moritz Wußing, Roland H. Grabner, Hannah Sommer, Henrik Saalbach

**Affiliations:** ^1^Faculty of Education, Leipzig University, Leipzig, Germany; ^2^Institute of Psychology, University of Graz, Graz, Austria

**Keywords:** language-switching costs, retrieval-based learning, encoding specificity, language dependency, subsequent learning, bilingual learning, mathematical learning

## Abstract

Language-switching costs arise when learners encode information in one language and subsequently recall that information in a different language. The assumed cognitive mechanism behind these costs is the principle of encoding specificity that implies language-dependent representations of information. The aim of our study was to test this mechanism and to gain insights into the impact of language-switching on subsequent learning. To this end, we used retrieval-based learning as a carrier-paradigm. In a 2×3-design, 117 participants learned mathematical concepts with a practice-test or a restudy opportunity (within-subjects factor). In addition, the sample was divided into three groups regarding language-switching (between-subjects factor): one group without switching, one switched for the final tests, and one switched between initial learning and subsequent learning. Results show the expected main effects: participants performed better for the items learned via retrieval-based learning (testing-effect) and worse in conditions with language-switching (language-switching-costs). Most importantly, we were able to find an interaction between learning condition and language-switching: retrieval-based learning suffers particularly from language-switching. Additionally, our results indicate that language switching before subsequent learning seems to be particularly detrimental. These results provide both validation for encoding specificity as mechanism underlying language-switching costs and new information on the impact of the time of language-switching that can be considered in educational designs such as “Content and Language Integrated Learning.”

## 1. Introduction

Bilingual learning and instruction approaches have gained widespread popularity in recent years (Pérez-Cañado, 2012). One of the most popular approaches is Content and Language Integrated-Learning (CLIL) where nonlinguistic subjects are taught in a language that students are still learning (see, e.g., Johnson and Swain, 1997; Swain and Lapkin, 2005). The promise of this educational approach is to promote competencies in both, a foreign language and a content-subject, at the same time (e.g., Dalton-Puffer, 2008). However, this educational approach may also come with cognitive costs which arise from switching between different languages across learning and retrieval (see, e.g., Saalbach et al., 2013; Volmer et al., 2018). The aim of the present study was to further examine these so-called language-switching costs (LSC). Specifically, we used *retrieval-based learning* as a carrier paradigm to investigate the impact of the time of language-switching on learning and to test the “*encoding-specificity hypothesis*” as a theoretical framework to explain LSC.

For bilingual education, researchers have repeatedly noted a severe imbalance between the popularity of bilingual approaches in education and its theoretical foundation (Wolff and Holmes, 2011; Goris et al., 2019). Even though numerous studies reported empirical benefits of bilingual education regarding language competencies, motivation as well as subject content knowledge (see Dalton-Puffer, 2008 for an overview), many studies indicated that students from bilingual education may perform worse or need more time to achieve the same level of knowledge compared to students in monolingual education (e.g., Lo and Lo, 2014; Dallinger et al., 2016; Piesche et al., 2016). In particular, several studies in recent years have reported language-switching-costs (LSC), reflecting worse performance or longer reaction times when knowledge is acquired in one language and subsequently retrieved in another (e.g., Marian and Fausey, 2006; Grabner et al., 2012; Saalbach et al., 2013) – a situation that may occur often in bilingual education. In typical empirical designs to investigate LSC, participants learn information in one language (L1) and have to retrieve this information either in the language the information was learned or in a different language (L2). In the latter case, LSC in terms of poorer performance were observed (e.g., Hahn et al., 2019). This effect has been reported in studies using a wide range of materials such as factual information about chemistry, biology or history (Marian and Fausey, 2006), autobiographical memories (Marian and Neisser, 2000) or – most widely employed – arithmetic knowledge (e.g., Spelke and Tsivkin, 2001; Saalbach et al., 2013) Moreover, neuroimaging studies revealed “qualitatively different brain responses” (Salillas and Wicha, 2012, p. 1) in conditions with language-switching compared to conditions without (Grabner et al., 2012).

LSC are associated to *language dependent knowledge representation* (Gentner and Goldin-Meadow, 2003; Kempert et al., 2019). According to this framework, information is not stored in a language-independent way but is closely bound to the language in which it was encoded. That language influences the representation of knowledge or its conceptual structure has also been shown by approaches comparing different languages. For instance, grammatical structures such as Chinese classifiers and German grammatical gender have been shown to affect how participants think about objects and draw inferences about properties (Saalbach and Imai, 2007; Saalbach et al., 2012). This language-dependence of knowledge representation has even been shown for mathematics, in general, and arithmetic, in particular (Van Rinsveld et al., 2015). For mathematics, this may come as a surprise, since one may argue that its content is highly abstract and numbers may be used without any language. However, there is plenty of evidence that language is indeed a crucial factor not just for students’ performance in mathematics at school (Kempert et al., 2011; Prediger et al., 2015) but also for performing basic arithmetic operations (Gordon, 2004; Pica et al., 2004).

A prominent theoretical framework to explain language dependent knowledge representations and LSC is the *encoding specificity hypothesis* (Tulving and Thomson, 1973). It proposes that during encoding the cognitive representation of an information is linked to context information that can later serve as *retrieval cue.* These cues, like *elaborations* in general, increase the embedding of the new information and hence their accessibility. Therefore, the effectiveness of retrieving knowledge depends on the similarity of the contexts of learning and retrieval. Even though this has been most often shown for the similarity of locations (Godden and Baddeley, 1975), language must be considered as a crucial part of the context as well. For instance, studies revealed that autobiographic memory of certain episodes is strongly bound to the language context in which they occurred (Matsumoto and Stanny, 2006). For bilingual learning settings, the encoding specificity hypothesis predicts that the retrieval and the application of knowledge is more effective in the language of encoding than in any other language (Marian and Neisser, 2000; Marian and Fausey, 2006) However, the empirical evidence of the encoding specificity hypothesis regarding language dependency in bilingual learning is still scarce.

Even less is known about the impact of language-dependent knowledge on subsequent learning of new information. There is rich evidence that prior knowledge is by far the strongest predictor for successful learning (Schwartz et al., 2005; Vosniadou, 2013). But to what extent does this hold when language switches throughout the learning processes? If access to language-dependent prior knowledge is hampered due to switching of the language of instruction, subsequent learning may also be impeded. To our knowledge, this potential educational challenge has not been examined so far. *Retrieval-based learning* may offer a unique opportunity to address the questions about subsequent learning and about the value of the encoding-specificity hypothesis to explain LSC.

Retrieval-based learning is a well-known and widely used educational tool to improve learning performance. Instead of teachers repeating information over and over again, learners are prompted to try to retrieve this information after an initial learning on their own. In fact, retrieval-based learning can be considered as the basis for any kind of exercises in which students retrieve information to solve the respective task. We expect retrieval-based learning to be especially vulnerable toward language-switching. Since LSC occur when information is retrieved in a different language after an initial learning, any learning design that explicitly builds on the process of retrieval should face the downsides of language-switching even more than other learning designs.

Retrieval-based learning has often been studied by means of the so called “Testing-Effect” (TE). TE refers to the phenomenon that retrieval-based learning is more effective than time-equivalent restudying (see, e.g., Rowland, 2014 for a review). Experimental designs usually present new information to all participants in an initial learning phase. Afterwards, participants either restudy the same information or complete a practice test on this information. After a certain retention interval, all participants take the same test in order to assess learning performance. Prior research revealed that the practice-test group significantly outperforms the restudy group after a certain retention interval (Carrier and Pashler, 1992; Toppino and Cohen, 2009). Effect sizes of the TE usually range between medium to large; for instance, Adesope et al. (2017) reported a mean effect size of *g* = 0.51 in their meta-analysis. In addition to this wide range of possible material, the TE also appears to be robust in transfer tasks. For example, Carpenter et al. (2006) showed that when word pairs A - B were learned, the testing effect also occurred when A – B was trained in the practice test, but B - A was asked in the final test. Pan and Rickard (2018) concluded in their meta-analysis that retrieval-based learning can lead to more successful transfer in general (weighted mean effect size: *d* = 0.40).

There is a wide range of theories providing various compatible theoretical explanations for the TE. Most commonly, it is attributed to the consolidation of language-dependent elaborations during retrieval (“*elaborative retrieval hypothesis*” – Carpenter, 2009). These elaborations are constructed during initial learning, and their use during the practice test improves future retrieval in the final test (Pyc and Rawson, 2010). This explanation is in line with the idea of language dependent knowledge representation that provides the general base on which LSC can be explained, since the formed elaborations cannot necessarily be accessed from a different language without additional cognitive load.

Another approach to explain the TE, the “*episodic context account*” (e.g., Lehman et al., 2014), assumes that episodic context information is built up during initial learning of an item (Karpicke et al., 2014) and will help future recall by acting as a retrieval cue. This information is then updated and supplemented to a test context when the item was retrieved in a practice test. Hence, in a final test, both the original and the updated episodic context information can support each other and thus improve recall performance. This latter approach obviously strongly overlaps with the *encoding specificity hypothesis* that is used to explain LSC.

A third approach is the idea of “*transfer appropriate processing*” (TAP) (e.g., Morris et al., 1977; Veltre et al., 2014). This approach suggests that overlapping cognitive processes in initial and final tests are responsible for the testing effect. So, increased levels of similarity between both tests should enhance the magnitude of the effect whereas the effect should be reduced if the two tests differ. However, this theoretical approach has received rather mixed empirical support (see, e.g., Carpenter and Delosh, 2006 for a study that did not find evidence for TAP or Rowland, 2014 for a meta-analytic overview).

All three lines of explanation for the TE suggest that the advantage of retrieval-based learning should be reduced when language switches during learning. On the one hand, language-dependent elaborations may only be consolidated when language of learning and language of retrieval are the same (*elaborative retrieval hypothesis*). On the other hand, episodic context information may be bound to a specific language and not be easily switched to a different language context (*encoding specificity hypothesis*). Regarding *transfer appropriate processing,* switching languages between the practice test and the final test reduces the similarity of both tests and hence the possibility to transfer overlapping processes to the final test.

## 2. The present study

The central aim of this study was to further examine LSC in bilingual mathematics learning. More specifically, we aimed to test the “encoding specificity hypothesis” as a possible framework for LSC (e.g., Marian and Fausey, 2006) and to examine the effect of language-switching on subsequent learning.

To this end, we applied retrieval-based learning as a carrier-paradigm. In a condition without language-switching, we expected to replicate the commonly reported finding of a higher solution-rate for items learned by retrieval than for items learned by restudying (see, e.g., Roediger and Karpicke, 2006a; Carpenter, 2009). We further expected information acquired by retrieval-based learning to be better applied to solve novel problems than information learned with restudying (Pan and Rickard, 2018). However, based on the theoretical considerations outlined above, in conditions with language-switching, we expected the beneficial effect of retrieval-based learning to be reduced.

To assess this expected pattern, we used 3 different between-subjects conditions: Condition 1 (“Monolingual”) served as a baseline-condition: no language-switching took place during the experiment, participants completed encoding, training, and final recall in L1. In condition 2 (“Switching for final tests”), participants switched from their L1 to their L2 after training, with the final assessment taking place in L2. This condition resembles classical designs to assess LSC (see, e.g., Marian and Fausey, 2006; Saalbach et al., 2013) in which information is completely encoded and trained in one language and subsequently assessed in another one. In condition 3 (“Switching for subsequent learning”), participants had to switch from L1 to L2 right in the middle of the learning process. They initially learned (encoded) the information in L1 but practiced or repeated them in L2 before they took the final test in L2 as well. This condition assesses the impact of language-switching on subsequent learning, a situation crucial in bilingual education: subsequent instruction or learning opportunities are provided in one language but are supposed to connect to information acquired in another language. Here, the access to language-dependent prior knowledge may be hampered because it is bound to a different language and subsequent learning may be impeded (e.g., Saalbach and Schalk, 2011).

Overall, we put forward the following hypotheses:

1. Language-switching between encoding and retrieval results in language-switching costs, i.e., lower accuracy when the language of encoding and retrieval differ compared to the condition in which they are the same. This effect should be larger when language-switching occurs during the learning process as compared to after learning.
2. We further expect to replicate the Testing-effect: solution rate in the retrieval-based learning condition is higher than the solution rate in the restudy condition.
3. There is an interaction effect between language-switching and learning condition reflecting that solution rate in the retrieval-based learning condition drops particularly strongly through language-switching compared to the solution rate in the restudy condition because the elaborations or episodic context information formed in one language may not be useful as cues for retrieval in another language.
4. All the hypotheses above can also be confirmed in a transfer test.

## 3. Methods

### 3.1. Participants

#### 3.1.1. Sample

One hundred seventeen adults aged between 19 and 31 years (*M* = 22.29, *SD* = 2.88) participated in this study. Sample size calculation was based on effect sizes reported for the two assumed main effects: language-switching costs (large effects around $\eta^{2}=0.19$ , e.g., Saalbach et al., 2013) and testing-effect (medium effects around *g* = 0.51, e.g., Rowland, 2014). Since the estimated necessary sample size was larger for the testing-effect, we used this value to carry out a power analysis with G*Power (Faul et al., 2007) and hence aimed for a sample of 40 students in each of the three conditions, so for a total sample of 120 participants. Since our study consisted of two sessions with 1 week in between, some participants dropped out by not completing the second session. This resulted in a final sample of 117 participants.

The sample mainly consisted of university students majoring mostly in psychology and teacher education. Participants were recruited in Leipzig and Graz via emails to designated distribution lists of the universities and via announcements on e-learning platforms. Since a sufficient level (B2) of English-proficiency is an enrollment-requirement for both universities, we could take this level for granted in our sample. Nevertheless, we controlled for the English-proficiency in a separate test. Participation was compensated monetarily or by partial course credit. The study was approved by the local ethics board of the University of Graz, following the tenets of the Declaration of Helsinki.

#### 3.1.2. Preliminary survey and exclusion criteria

In order to test only individuals suitable for the actual study, an online pre-survey was administered.

After general information about the study, the pre-survey included a demographic data questionnaire. Subsequently, prior knowledge in the area of the information that had to be learned in our study, was evaluated on a Likert scale from 1 to 10. Finally, potential participants completed the LexTale to assess their level of English. The LexTale serves as a quick and reliable measure to assess a person’s language ability (Lemhöfer and Boersma, 2012). In this test, a decision must be made for 60 words as to whether or not a given word exists in English.

We only invited participants to the actual study that had no learning disorders, were aged between 18 and 35, did not rate their prior knowledge in the relevant areas with 5 (out of 10) or above and reached a LexTale score of 50 (out of 100) or above. This limit of 50 for the LexTale score was chosen as one standard deviation below the mean reported by Lemhöfer and Boersma (2012).

### 3.2. Material

#### 3.2.1. Learning material

The learning material of our study consisted of two texts (found as supplementary material, see Data Availability statement) that introduced and explained mathematical terms and concepts. The first text was about propositional logic and consisted of 258 words, the second one was about mathematical functions (“mappings”) and consisted of 276 words. Each text consisted of 10 passages of approximately equal length that explained one single concept such as the idea of a “bijective function” or a “tautology “by 18 to 33 words. To enable understanding of the material in the language-switching condition, target concepts terms such as “surjective” (= “surjektiv” in German) or “negation” (= “Negation” in German) were chosen to be as similar as possible in both languages.

We designed these texts to provide realistic mathematical learning material that was novel to potential participants and contained both fact learning and conceptual insight. Presenting short texts is a common procedure to study both the testing-effect (Roediger and Karpicke, 2006a) and language-switching costs (Marian and Fausey, 2006). The texts were originally written in German and were translated into English by the test administrators and subsequently checked and corrected by two native speakers.

#### 3.2.2. Assessment tools

For the final test, we designed both a cued-recall and a transfer test for each of the two texts.

In the cued recall test, the terms of the texts had to be reproduced based on a short explanation of the concept. Even though these explanations were similar to the ones used in the texts itself or during the practice test, they were not identical. In case of minor deviations of the participants’ spelling from the correct spelling of the terms, we manually recoded answers to be counted as correct. For this purpose, both test administrators checked all the given answers individually for answers that had to be recoded and reached an inter-rater reliability of 100%.

Finally, there was a transfer test for each text, in which the concepts had to be applied to concrete working examples. Participants were presented 20 short mathematical problems and associated statements (10 for each text) and had to decide for each statement whether it was correct or incorrect.

Moreover, a 5-min verbal distractor task was designed to be used between the initial learning phase and the intervention phase. Participants were instructed to write down as many words as possible with three given initial letters (0,40 min each) in an empty input window of the stimulus presentation, one letter at a time. The paradigms for study and test trials were programmed using the software *PsychoPy* (Peirce et al., 2019) and supplemented with *LimeSurvey* polls (Limesurvey GmbH) for initial instructions and questionnaires.

### 3.3. Procedure

#### 3.3.1. General paradigm

Participants who matched the criteria in the pre-survey underwent a two-day training study with a retention interval of 7 days. Each of the two sessions took about 35–40 min and was completed in computer labs of Graz or Leipzig University.

On the first day, all participants completed an initial learning phase, in which the two texts were presented for 150 s each. The order of the texts was counterbalanced. There were 2 runs of this initial learning phase to provide a sufficient level of knowledge after this phase. Afterwards, participants completed the distractor-task on verbal fluency. In the following intervention phase, one of the texts was repeatedly presented in a restudy option, the other one was tested in a practice test. The assignment of the texts to one of these options was counterbalanced. In both cases, the texts were split into the 10 paragraphs containing the single concepts. In the restudy option, these passages were presented for 20 s each. In the practice-test option, the concepts names had been deleted and replaced by a blank line. Participants were prompted to fill in the name of the missing concept within 18 s for each of the 10 concepts. After time had passed for each concept, feedback was displayed for 2 s, which contained the information about the correctness of the solution as well as the correct solution itself on green (correct solution entered) or red (incorrect solution entered) background. In this vein, the texts in both conditions received equal exposure time. This entire intervention phase of alternating restudy condition and test condition was also repeated twice to ensure a sufficient level of knowledge.

After a retention interval of 7 days, the participants completed the second part of the study. Here, we administered two final tests: a cued recall test and a transfer test (see *Assessment tools*). In the cued recall test, participants were instructed to enter the correct concepts name to the presented description and confirm their answer within 30 s for each concept. No feedback was provided during this test. Immediately afterwards, a transfer test (see *Assessment tools*) was conducted. In this test, participants had to evaluate the correctness of a given statement within 30 s for each of the 20 questions. Since the testing-effect is expected to be effective only after a certain retention interval (Toppino and Cohen, 2009), we expected this second final test to be only marginally influenced by the first final test.

#### 3.3.2. Language-switching

**Figure 1:**
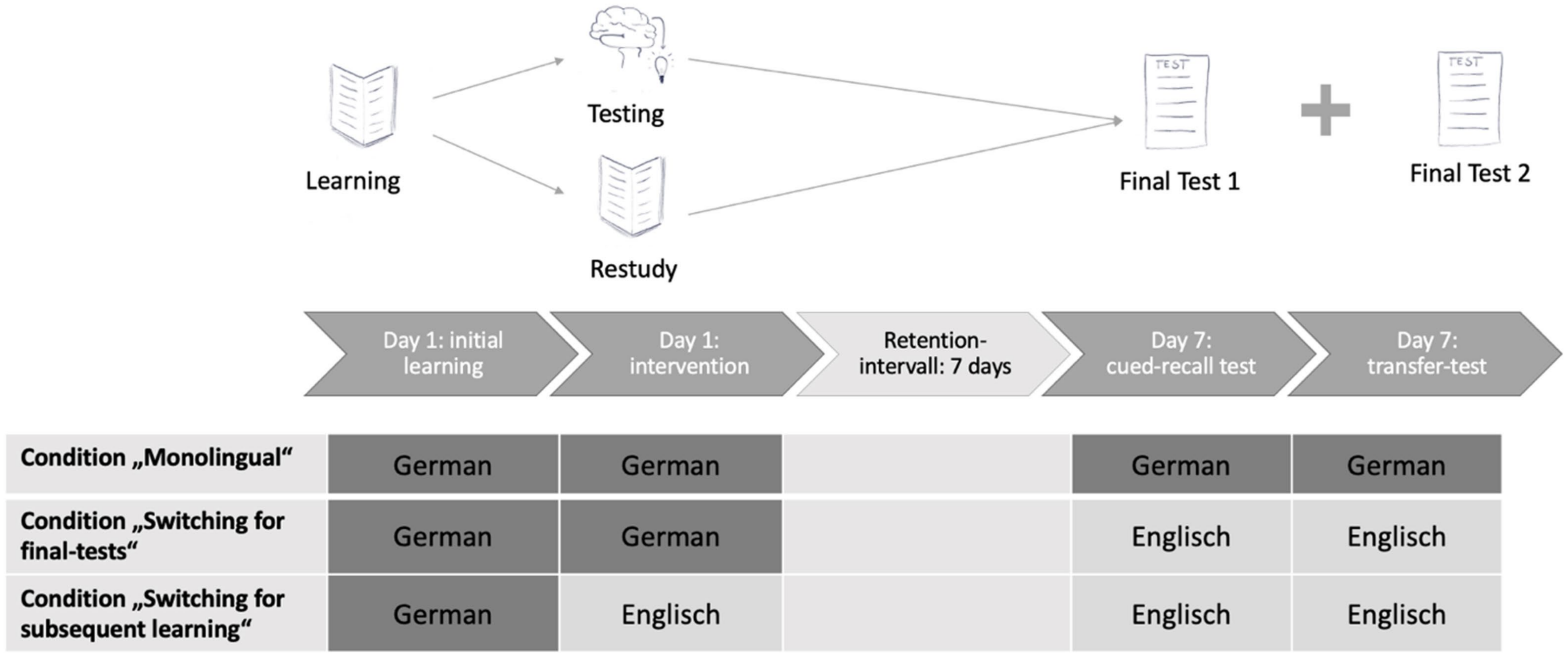
Schematic overview of the three between-subjects conditions regarding language-switching.

The design presented above was carried out in three different conditions regarding language-switching. A schematic representation is displayed in Figure 1. In the first condition, “Monolingual,” no language switching was necessary. All three phases of the experiment (initial learning, training, final testing) were conducted in German. In condition 2, “Switching for final tests,” the first two phases were conducted in German, but both of the final tests (cued-recall and transfer) were carried out in English. Hence, participants learned and trained information in one language but had to retrieve them in another one. This condition represents the classical design to assess LSC, where information is learned in one language and retrieved in a different one. In the third condition, “Switching for subsequent learning,” however, language-switching took place directly after the initial learning phase. The initial learning was carried out in German, but the training (one text by restudying, one text by testing) and the final tests were carried out in English. Hence, in this condition switching took place during the learning process itself, between the initial learning and the subsequent learning in form of training (either restudying or retrieval-based learning).

We deliberately decided against conditions using English as learning-language. We focused on the interaction between LSC and the TE. The directionality of LSC was not part of our questions, since previous studies (Saalbach et al., 2013; Hahn et al., 2019) were able to show, that LSC arise for both directions of switching: from L1 to L2 and from L2 to L1.

#### 3.3.3. Data analysis

The statistical analysis was conducted by computing repeated measures ANOVAs for each of the final tests. Our within-subjects factor *Learning condition* included two levels: restudying or testing. The between-subjects factor *Language-switching* included three levels: no switching (“Monolingual”), “switching for the final tests” and “switching for subsequent learning.” Post-hoc tests using Bonferroni-corrections were computed for significant effects.

## 4. Results

### 4.1. Pre-analyses of the training test

First, we analyzed performance data of the training test during the intervention phase. Note that in this test only items in the respective testing-conditions can be considered. In general, participants solved 3.80 (out of 10, SD = 2.68) items correctly in the first practice test. As expected, in the second practice-tests, this number significantly increased to 5.75 (SD = 2.92).

We also analyzed differences between the conditions in training test performance. An ANOVA revealed a significant difference between the conditions [*F*(2,115) = 10.087, *p* < 0.001, $\eta^{2}=0.150$ ]. *Post hoc* tests showed no difference between the conditions “Monolingual” (solution rate = 45.25%), and “Switching for final-test” (solution rate = 45.26%), but for the condition “Switching for subsequent learning” (solution rate = 23.33%). This result is expectable, given the fact that only the initial test in this condition included language-switching.

### 4.2. Analysis of cued-recall test

**Figure 2:**
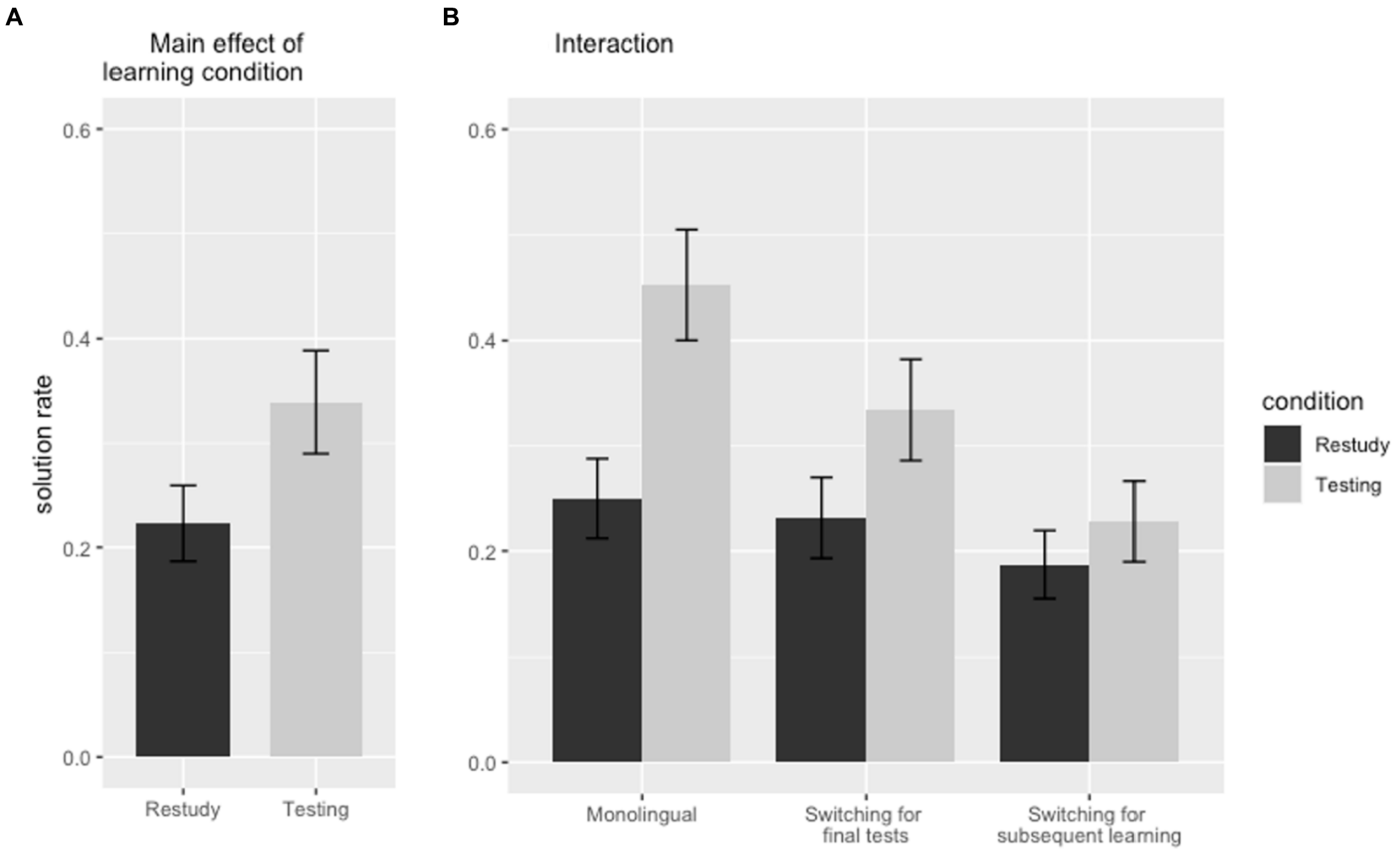
Results of the cued-recall test, Error bars represent the 95% confidence interval. **(A)** Main effect of learning condition, **(B)** Interaction between learning condition and language-switching.

We analyzed performance data of the cued-recall test of all 117 participants. The overall solution rate for this test was 5.624 (out of 20) with a standard-deviation of 4.087. An overview of the results of the cued-recall test is displayed in Figure 2. The ANOVA for the cued-recall test revealed a main-effect of learning condition [*F*(1,116) = 29.224, *p* < 0.001, $\eta^{2}=0.057$ ]. Items that were learned with the use of a practice test (*M* = 3.393 out of 10, *SD* = 2.691) were remembered better than items learned via restudying (*M* = 2.231, *SD* = 1.989). This result is a replication of the well-established testing-effect, i.e., the benefit of retrieval-based learning, and in line with our first hypothesis.

Furthermore, we found a second main effect for language-switching [*F*(2, 115) = 5.231 *p* = 0.007, $\eta^{2}=0.059$ ]. Performance in the monolingual condition (*M* = 7.025 out of 20, *SD* = 4.123) was significantly better than in the two conditions including language-switching (*M* = 4.896, *SD* = 3.899). This result is a further confirmation of the existence of LSC and supports our second hypothesis. *Post hoc* tests further helped to narrow down the decline in performance. A significant difference in performance was observed only between conditions “Monolingual” and “Switching for subsequent learning” (*p* < 0.001, *d* = 0.768) but not between conditions “Monolingual” and “Switching for final tests” (*p* = 0.158, *d* = 0.323). This result highlights the special impact of language-switching on subsequent learning.

As hypothesized, there was also a significant interaction between learning condition and language-switching [*F*(2,115) = 13.132, *p* = 0.009, $\eta^{2}=0.019$ ]. With regards to the testing-effect in the individual conditions, the benefit of retrieval-based learning was found in “Monolingual” [*t*(39) = 4.550, *p* < 0.001, *d* = 0.719], to a weaker extent in “Switching for final tests” [*t*(37) = 3.264, *p* = 0.002, *d* = 0.530] and not at all in “Switching for subsequent learning” [*t*(38) = 1.251, *p* = 0.219, *d* = 0.200]. The decline of the benefit of retrieval-based learning was found to be significant between the monolingual condition and the “Switching for subsequent learning” [*t*(77) = 2.909, *p* = 0.005, *d* = 0.655]. Between “Monolingual” and “Switching for final tests,” the decline of the benefit or retrieval-based learning did not reach significance [*t*(76) = 2.909, *p* = 0.073, *d* = 0.411]. A further analysis for the differences between conditions “Monolingual” and “Switching for subsequent learning,” split for items learned via restudying and via retrieval, supports our assumption that LSC arise from a particular weakening of retrieval-based learning: a significant difference only emerged for the items learned via retrieval-based learning (*p* < 0.001, *d* = 0.893), but not for the items learned via restudying (*p* = 0.152, *d* = 0.326).

### 4.3. Analysis of transfer test

The overall accuracy of the transfer test is at 13.529 items (out of 20) with a standard deviation of 2.420. Note that this seemingly higher solution rate is biased by the yes/no-choice design of this test. However, a one sample t-test marked a significant deviation from pure guessing [t(116) = 15.741, *p* < 0.001, *d* = 1.455].^1^ Contrary to our predictions, results in the transfer test differed clearly from the results in the cued-recall test.

First, the main effect of learning condition was not significant [*F*(1,116) = 1.827, *p* = 0.205, $\eta^{2}=-0.007$ ]. Participants solved 6.607 (*SD* = 1.814) of the 10 questions covering concepts learned via testing and 6.915 (*SD* = 1.798) of the questions covering concepts learned with restudying. Hence, we were unable to replicate the assumed transfer of the testing-effect. To reduce the possible distorting influence of language-switching, we analyzed only the results from condition 1 (no language-switching), but the absence of a significant effect remained (*p* = 0.681, *d* = 0.066). However, in the condition “Switching for subsequent learning,” a significant difference was observed. Participants solved 5.821 (*SD* = 1.715) of the 10 items learned via retrieval and 7.154 (*SD* = 1.598) of the 10 items learned via restudying [*t*(38) = −3.570, *p* < 0.001, *d* = −0.572] – this effect was indeed significant, but in favor of restudying, which represents an inverse testing-effect.

Second, regarding the main-effect of language-switching, participants solved 13.975 out of 20 items (*SD* = 2.616) in the condition without language-switching and 13.286 items (*SD* = 2.293) in the conditions with language-switching. But this expected main-effect for language-switching was not significant [*F*(2,115) = 1.744, *p* = 0.179, $\eta^{2}=0.013$ ]. Therefore, no post-hoc analysis were computed for the effect.

**Figure 3:**
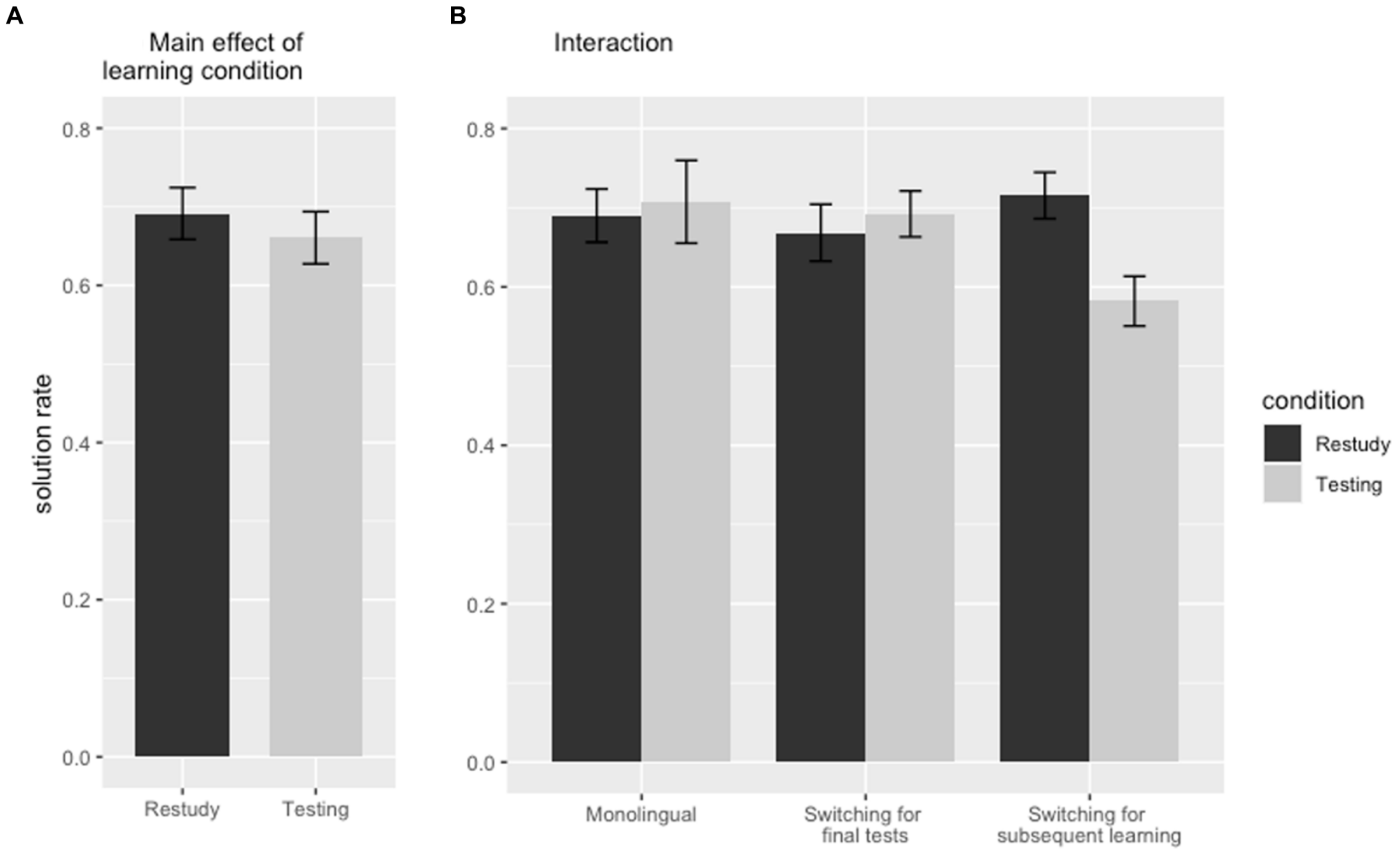
Results of the transfer test. Error bars represent the 95% confidence interval. **(A)** Main effect of learning condition, **(B)** interaction between learning condition and language-switching.

However, the interaction between the two main effects was still significant [*F*(2,115) = 4.541, *p* = 0.013, $\eta^{2}=0.040$ ]: the efficiency of retrieval-based learning seems to be influenced by language-switching. Post-hoc analyses were carried out for this interaction: a significant difference was only observed for the items learned via retrieval between the conditions “Monolingual” and “Switching for subsequent learning” (*p* = 0.003 *d* = 0.693). All other differences remained insignificant – especially all differences in items learned via restudying. This result may be seen as a further indication that LSC arise from a particular weakening of retrieval-based learning (Figure 3).

## 5. Discussion

The main goal of our study was to further examine language-switching costs (LSC) by using retrieval-based learning as a carrier paradigm. Specifically, we aimed to test the encoding-specificity hypothesis as an underlying mechanism of LSC and to gain insights into the influence of the position of language-switching during the learning process. To this end, participants underwent a training study in the general design commonly used to evaluate the advantage of retrieval-based learning (testing-effect). This design was carried out in three different conditions regarding language-switching: one condition without language-switching, one condition with language-switching right before the final test and one condition with switching between the initial learning and the training (subsequent learning). We analyzed the performance data of two final tests: a cued-recall test and a transfer test.

As predicted, two main-effects were observed in the cued-recall test. First, participants showed higher accuracy when having learned the items using a practice test (i.e., retrieval-based learning) than when having learned the items through an additional restudy session. The size of this effect over all conditions ( $\eta^{2}=0.058$ – small to medium effect) is slightly smaller than the expected effect size for the testing-effect (*g* = 0.51, Adesope et al., 2017 – medium effect). This is most probably due to language-switching in two of the three conditions. In fact, when only considering data from the no language-switching condition, the effect size is even larger than what we initially expected (*d* = 0.719). This result is a further proof of the benefit of retrieval-based learning and hence its relevance for education in general (Roediger and Karpicke, 2006b; Agarwal et al., 2009).

Second, language-switching reduced participants’ performance: participants showed lower accuracy when they had to switch languages during the learning process (“Switching for subsequent learning) or for the recall of information (“Switching for the final-tests”) compared to a condition, where no language-switching was required. This outcome is a replication of previous findings regarding LSC (Grabner et al., 2012; Saalbach et al., 2013). However, our data allowed us to further narrow down the occurrence of these costs. Post-hoc analysis showed a significant decrease of performance only between “Monolingual” and “Switching for subsequent learning” (*d* = 0.299), not between the monolingual condition and “Switching for final tests” (*d* = 0.155). This result was unexpected given prior research. Previous studies on LSC used paradigms that resembled the design of condition 2: participants studied the learning material in one language completely before they retrieved them in a final test in the other language (e.g., Marian and Fausey, 2006). Even though LSC were repeatedly found using this design, we were unable to reveal a significant decline in performance in this condition. In our study, LSC only occurred when language-switching takes place within the learning process itself (“Switching for subsequent learning”). This finding highlights the relevance of the exact timing of language-switching during the process of learning.

Third and most importantly, we observed an interaction between learning condition and language-switching. The advantage of retrieval-based learning is influenced by language-switching. Even though this interaction was significant over all conditions, post-hoc tests marked the interaction between several single conditions as non-significant, including all conditions with items learned via restudying. This result supports our assumption that dominantly a downfall in the performance in the testing condition is responsible for the general decline of performance in the conditions with language-switching - which is in line with our third hypothesis: retrieval-based learning suffers particularly from language-switching. This result indicates that the usability of elaborations (Carpenter, 2009) or additional episodic context information (Lehman et al., 2014) seems to be impeded by language-switching, so that the advantage of retrieval-based learning is reduced. Therefore, our results support the “encoding specificity hypothesis” (Tulving and Thomson, 1973; Marian and Fausey, 2006) as an explanation of LSC: the specific episodic context of encoding and retrieval does not match and hence the formed retrieval cues cannot be easily accessed. Information seems to be represented in a language-dependent way (Gentner and Goldin-Meadow, 2003 Kempert et al., 2019) since the access to additional context information that could facilitate their recall, is probably bound to a specific language.

Additionally, the post-hoc analysis allowed us to further narrow down the interaction to a difference in the items learned via retrieval between the conditions “Monolingual” and “Switching for subsequent learning” (*p*_bonf_ < 0.001) but not between the monolingual condition and “Switching for final tests.” We can derive a new and interesting information about the impact of language-switching on subsequent learning from this result. Designs, where subsequent learning takes place in a different language, are even more vulnerable to language-switching than designs that require language-switching only for the final assessment. In other words: learning that builds on previous information available in a specific language – which is usually the case in everyday mathematical curriculum – may face even more challenges regarding language-switching than previous studies indicated that demanded for switching only for the final assessment (Marian and Fausey, 2006; Saalbach et al., 2013). Therefore, this finding highlights the importance of considering issues arising from language-switching in bilingual learning settings.

The lack of a significant decrease of the retrieval-based learning advantage between the monolingual and the “Switching for final tests” condition could be considered as an additional interesting hint on the nature of LSC: As outlined above, we assume LSC to be based on a reduced usability of retrieval cues or context-information, which are known to be improved by retrieval-based learning (e.g., Carpenter, 2009). Pyc and Rawson (2010) suggested that such cues are formed during initial learning, consolidated and flexibilized during retrieval, so that they can be better used during final recall. We initially expected the usability of the language-dependent elaborations during the final recall to be reduced if participants had to use them in a different language (“Switching for subsequent learning”). However, we observed the strongest decline in performance when participants were disturbed in the *consolidation* of the elaborations due to the language-switching in the “Switching for subsequent learning” condition right after the initial learning phase. This indicates that LSC may not only arise from a reduced access to language dependent context information in the case of language-switching, but also from an impaired consolidation of such retrieval cues when language-switching occurs between initial and subsequent learning.

Finally, in our results of the transfer test, neither of the assumed main effects (testing-effect or language-switching costs) reached significance. For the testing-effect, this result differs from previous research (e.g., Rohrer et al., 2010; Carpenter, 2012; Pan and Rickard, 2018). Even in our baseline-condition without language-switching, we could not find a significant testing-effect in the transfer test. One may argue that performance in the transfer test may be biased by administering the first final test which also represents a retrieval opportunity. However, given that testing is known to cause benefits only after a certain retention interval (Roediger and Karpicke, 2006a), this explanation may not hold. More importantly, a closer look at previous studies reveals (as concluded for example by Tran et al., 2014) that most of the studies showing an improved performance in a transfer test for retrieval-based learning were administered with near-transfer items such as a change between cued recall and free recall (Kang et al., 2007) or a transfer in the stimulus–response arrangement (Carpenter et al., 2006). Studies that examined far-transfer such as application to new contexts (e.g., Wooldridge et al., 2014) or complex problems (Leahy et al., 2015) did not report significant advantages for retrieval-based learning. Hence, our results further indicate that far-transfer of retrieval-based learning should not be taken for granted and further research is strongly necessary for mathematical contexts. However, the interaction between language-switching and retrieval-based learning was still observed for transfer which is consistent with our assumptions.

Our study is subject to several limitations. First, we did not include conditions, where participants initially learned in English. Hence, our study does not contribute answers to the question whether LSC are bound to a specific direction (L1 to L2 or L2 to L1). However, since previous studies provided evidence for LSC in both directions (Saalbach et al., 2013; Hahn et al., 2019), we did not include this research question in the present study.

Second, our design caused a reduced initial-test performance in the group “Switching for subsequent learning.” This lower initial-test performance may have caused the reduced final-test performance in the condition “switching for subsequent learning.” However, this is in line with our expectations and is indeed a further piece of evidence for the existence of LSC. Furthermore, this reduced initial test performance cannot explain the reduced TE we observed. The meta-analysis of Rowland (2014) suggests that, as long as feedback is provided, a low initial-test performance should cause an even larger TE. That is because of the assumption that “desirable difficulties” (Bjork, 1994) during the practice test are beneficial for the TE. Therefore, in our opinion, the observed differences in the initial-test performances do not interfere with our conclusions.

Third, the lack of significance regarding the TE of the cued recall test in the condition “Switching for subsequent learning” may be due to a lack of power. That is because we based our power-analysis on effect-sizes of studies without moderating factors such as language-switching that could reduce the TE. Future studies should therefore consider aiming for a larger sample.

Lastly, we have to acknowledge that the self-assessment questions we used regarding prior knowledge in the area of our material may not have provided a satisfyingly objective measurement. Future studies should include a more detailed assessment of the mathematical abilities to both control for possible distortions and to use the mathematical ability to assess individual differences, which are highly important for education.

## 6. Conclusion

The present study provided further evidence for LSC in demanding mathematical tasks as well as information to better understand the cognitive mechanisms underlying LSC. Our study suggests that retrieval-based learning is particularly weakened by language-switching. Based on the overlapping theories behind LSC and retrieval-based learning, our findings can be seen as a validation of the encoding-specificity theory as an explanation for LSC. Furthermore, we found that language-switching within the learning process may cause even more challenges than switching languages between learning and testing. These insights may be crucial for designing bilingual educational settings in the future.

## Data availability statement

The datasets presented in this study can be found in online repositories. The names of the repository/repositories and accession number(s) can be found at: https://osf.io/83bqs/?view_only=1897bac456db4495b2a7998428dfaa73.

## Ethics statement

The studies involving human participants were reviewed and approved by Universität Graz Ethikkommission. The patients/participants provided their written informed consent to participate in this study.

## Author contributions

MW, RG, HaS, and HeS contributed to conception and design of the study. MW and HaS administered the test-sessions and performed the statistical analysis. MW wrote the first draft of the manuscript. All authors contributed to manuscript revision, read, and approved the submitted version.

## Conflict of interest

The authors declare that the research was conducted in the absence of any commercial or financial relationships that could be construed as a potential conflict of interest.

## Publisher’s note

All claims expressed in this article are solely those of the authors and do not necessarily represent those of their affiliated organizations, or those of the publisher, the editors and the reviewers. Any product that may be evaluated in this article, or claim that may be made by its manufacturer, is not guaranteed or endorsed by the publisher.

## Footnotes

^1^Due to the low solution rate, we also conducted all analyses concerning the transfer test excluding participants that performed at chance level or below (*n* = 8 in total, 3 in condition “Monolingual,” 1 in condition “Switching fur final tests” and 4 in condition “Switching for subsequent learning”). We did not observe any major changes in the results, so we only report the analyses including all participants.
